# Accelerators for Classical Molecular Dynamics Simulations of Biomolecules

**DOI:** 10.1021/acs.jctc.1c01214

**Published:** 2022-06-16

**Authors:** Derek Jones, Jonathan E. Allen, Yue Yang, William F. Drew Bennett, Maya Gokhale, Niema Moshiri, Tajana S. Rosing

**Affiliations:** † Department of Computer Science and Engineering, University of California, San Diego, 9500 Gilman Drive, La Jolla, California 92093, United States; ‡ Global Security Computing Applications Division, Lawrence Livermore National Laboratory, 7000 East Avenue, Livermore, California 94550, United States; § Biosciences and Biotechnology Division, Lawrence Livermore National Laboratory, 7000 East Avenue, Livermore, California 94550, United States; ∥ Center for Applied Scientific Computing, Lawrence Livermore National Laboratory, 7000 East Avenue, Livermore, California 94550, United States

## Abstract

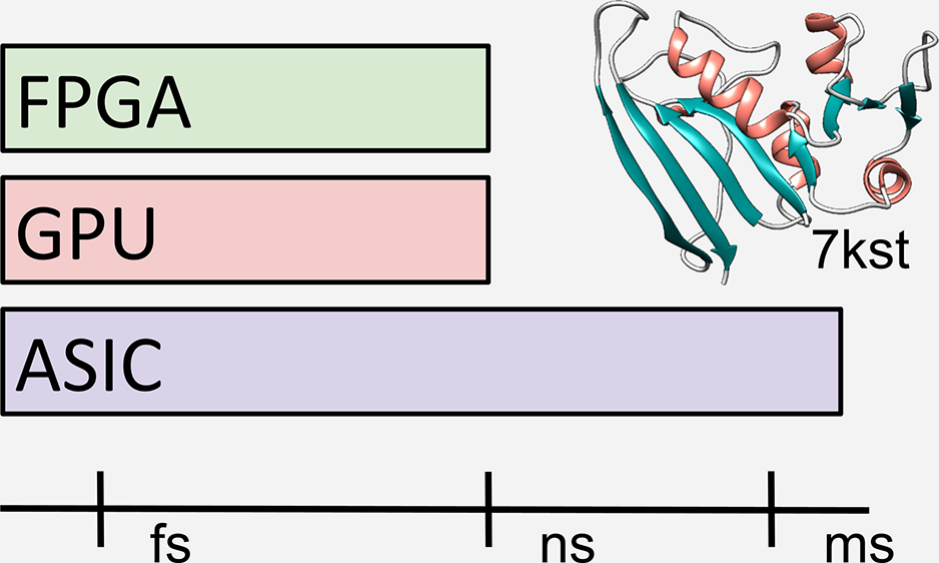

Atomistic Molecular Dynamics (MD) simulations provide researchers the ability to model biomolecular structures such as proteins and their interactions with drug-like small molecules with greater spatiotemporal resolution than is otherwise possible using experimental methods. MD simulations are notoriously expensive computational endeavors that have traditionally required massive investment in specialized hardware to access biologically relevant spatiotemporal scales. Our goal is to summarize the fundamental algorithms that are employed in the literature to then highlight the challenges that have affected accelerator implementations in practice. We consider three broad categories of accelerators: Graphics Processing Units (GPUs), Field-Programmable Gate Arrays (FPGAs), and Application Specific Integrated Circuits (ASICs). These categories are comparatively studied to facilitate discussion of their relative trade-offs and to gain context for the current state of the art. We conclude by providing insights into the potential of emerging hardware platforms and algorithms for MD.

## 1 Introduction

Now more than ever, the SARS-CoV-2 pandemic demonstrates the need to rapidly design therapeutic treatments to protect against diseases that pose a grave threat to human health.^1^ It is well-known that drug design remains an expensive and inefficient process consuming nearly a decade of time on average with total costs regularly cited in the billions of USD to develop a single successful candidate.^2,3^ Compounding this issue is the fact that the “drug-like” chemical space is itself not well understood as a whole, with estimates of the upper bound on this space varying between 10^18^–10^100^ depending on the assumptions made.^4^ While modern purchasable compound libraries only cover a small fraction of the prospective drug-like chemical space, it is now possible to commercially order molecules from a library of 10s of billions of virtual compounds.^5,6^ Thus, the scale of molecular interrogation needed in drug discovery necessitates alternatives to purely experimental approaches.

Classical Atomistic Molecular Dynamics (MD) simulations provide researchers the ability to apply a “computational microscope” to study the dynamic properties of biomolecules at spatiotemporal scales that current experimental methods are not able to access.^7^ The dynamics of interest include protein folding, protein-drug binding, conformational change, and transmembrane transport of substrates.^7^ MD as a field of study has existed for well over 70 years, with some of the first works reported in the late 1950s and early 1960s, though it was not until 1977 that the first MD simulation of a protein (BPTI) was considered.^8−10^

MD simulations are also notoriously expensive computational endeavors. By repeatedly integrating Newton’s equations of motion over very small timesteps, trillions of iterations are required before biologically relevant time scales can be reached. In the face of these challenges, hardware accelerators have played a crucial role in allowing MD simulations to unveil biological phenomena that would otherwise be practically infeasible using traditional hardware architectures.^11^ Over the years, accelerators for MD have grown from *Application Specific Integrated Circuits* (ASICs) to now include *Graphics Processing Units* (GPUs) and *Field Programmable Gate Arrays* (FPGAs). As a product of the end of Moore’s law, richer programming development environments, and algorithmic advances, biologically relevant simulation time scales are becoming more readily available to researchers without access to specialized architectures or High-Performance Computing (HPC) resources.

The goal of this perspective is to review MD hardware acceleration work to facilitate a comparative discussion on current approaches and gain insight into opportunities to improve on the current state of the art. Industry trends suggest that future hardware environments will be heterogeneous motivating the need for a review that explores the trade-offs of different hardware acceleration approaches. The structure of this article is as follows:

- We will provide an overview of the MD algorithm and its components in Section 2 to provide a sufficient level of background information for the latter sections. We then discuss the algorithms employed for the primary bottleneck in MD simulations, nonbonded force computations, in Section 3.
- Following the coverage of the algorithms, we discuss the implementations of MD engines for various hardware acceleration platforms. We begin this by discussing GPU-based MD acceleration in Section 4. GPUs have been increasingly employed for MD simulation given their successes in applications requiring intensive mathematical operations such as matrix multiplication (for example, Deep Learning). We briefly discuss the history of GPU-based MD engines and then discuss current limitations as well as open areas for research.
- FPGA-based MD acceleration work is discussed in Section 5. The discussion is split into three subsections; beginning with the early developments in the area, we then describe the subsequent push toward production-focused MD engines followed by recent developments in FPGA-based MD engines, concluding with a comparative analysis of these three distinct periods.
- The final class of ASIC-based architectures is presented in Section 6 where we discuss the early work in the area followed by the two prominent ASIC architectures; Anton by the D.E. Shaw Research group and the MD-GRAPE project by the Riken Institute.
- We then conclude with a comparative discussion of the trade-offs for each class of architectures and expand upon future directions for research in MD acceleration.

## 2 Overview of Molecular Dynamics Algorithm

### 2.1 Algorithm Description

**Figure 1:**
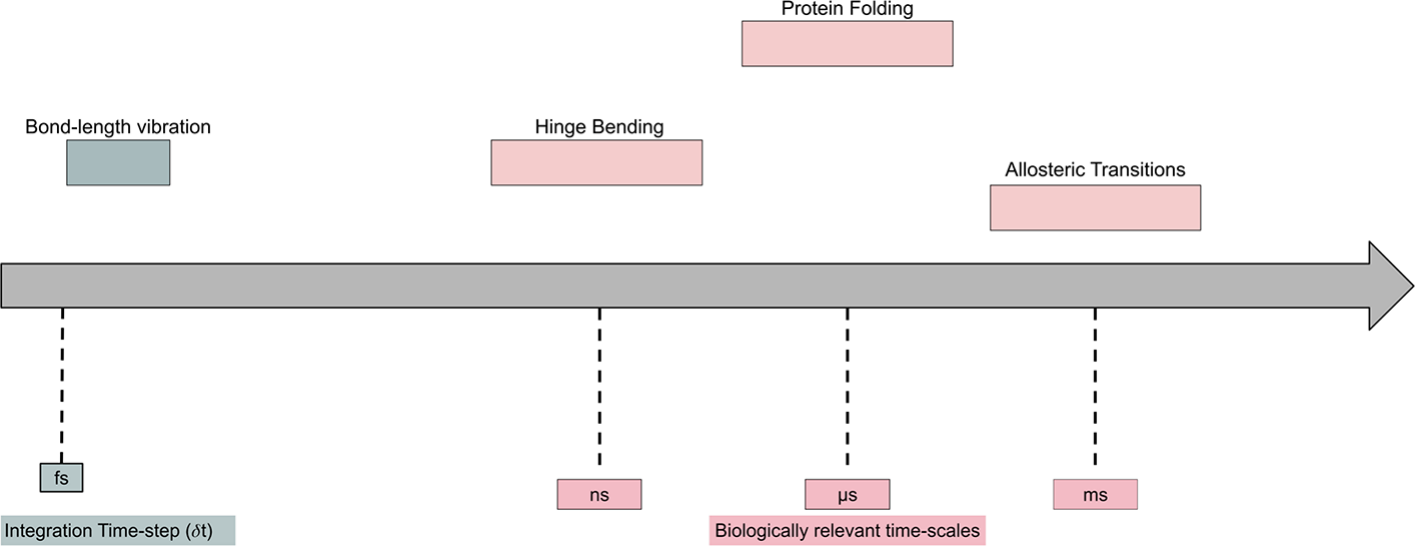
Representative time scales for protein motions.^12,15−17^

Molecular Dynamics (MD)^7,12−14^ considers an “*n*-body” system of particles (that is, atoms) where each particle possibly exerts a nonzero force on all of the other particles in the system. A description of the relevant biological events is given in Figure 1.

At each iteration, bonded interactions are first computed. The number of bonded interactions tends to be relatively small, thus this step can be done in time proportional to *O*(*n*) with *n* being the number of atoms. In contrast to the subsequent nonbonded step, the number of interactions can grow to be proportional to *O*(*n*^2^) in the worst case when considering all pairwise combinations. Afterward, the acceleration vectors for each atom are updated followed by their positions, completing one iteration of the simulation loop.

Time steps that are chosen for simulations are typically on the order of femtoseconds (10^–15^s) for reasons concerning numerical stability and simulation quality. The magnitude of the timesteps are consequently very small in comparison to the biologically relevant time scales.^17,18^ The number of sequential operations needed to achieve these time scales can grow to be at least 10^9^–10^12^.^7^

### 2.2 Force Computation

The first ingredient of an atomistic MD simulation of *n* atoms are their positions in Euclidean space, given as  for the *i*^th^ atom. The full set of *n* atom positions is given by the vector *r*. Each of the *n* atoms additionally carries a charge given by *q*_*i*_. Potential energy of the system, given as *U*, can then be computed using *r*_*i*_ and *q*_*i*_ for each of the *n* atoms. The specific form of the potential energy function can vary (for example, refs (19, 20, and 21)), the model we consider gives a basic idea of the components.^20^

The forces on each atom are then defined as the negative gradient of the potential energy function:
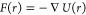
1where *F* is a vector-valued function, *r* is a position vector corresponding to a specific atom in the simulation.

The potential energy function that we consider within the context of molecular dynamics simulations is generally defined as
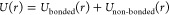
2which decomposes the potential energy function into the sum of bonded and nonbonded terms.

#### 2.2.1 Bonded Interactions

The bonded interactions can be further decomposed into the spring potential between atom pairs separated by a single covalent bond (1,2-pairs), the angular bond potential, and the torsion angular potential between atoms connected by 3 covalent bonds (1,4-pairs).
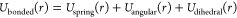
3

The term *U*_spring_ describes the bonded potential energy of 1,2-pairs as a function of the displacement of the bond length from its equilibrium position and is defined as
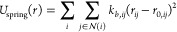
4where  denotes the set of *j* indices with covalent bonds to atom *i*, *k*_*b*,*ij*_ is the spring constant describing the strength of the bond, *r*_0,*ij*_ is the equilibrium bond length, where both *k*_*b*,*ij*_ and *r*_0,*ij*_ are specific to the atom types of the 1,2-pair, and *r*_*ij*_ = ||*r*_*i*_ – *r*_*j*_||.

Subsequently, *U*_angular_ describes the movement of bond angles from their equilibrium positions is defined as
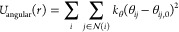
5where θ_*ij*_ is the angle between vectors *r*_*ij*_ = *r*_*j*_ – *r*_*i*_ and *r*_*kj*_ = *r*_*j*_ – *r*_*k*_, θ_*ij*,0_ is the equilibrium angle, and *k*_θ_ is the angle constant.

Finally, the 4-body torsion angle potential *U*_dihedral_ models the presence of steric barriers between the planes formed by the first three and last three atoms of a consecutively bonded (*i*, *j*, *k*, *l*)-quadruple of atoms (that is, 1,4 pairs):
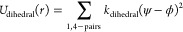
6where ψ is the angle between the (*i*, *j*, *k*)-plane and the (*j*, *k*, *l*)-plane, ϕ is the equilibrium angle between the two planes, and *k*_dihedral_ is a constant. The exact form of the 4-body torsion potential varies between force field definitions.

#### 2.2.2 Non-Covalent Interactions

While covalent interactions occur over relatively short distances (approximately 1–2 Å), noncovalent interactions may occur over much larger distances that could in theory involve any pair of atoms in the full simulation space.

**Figure 2:**
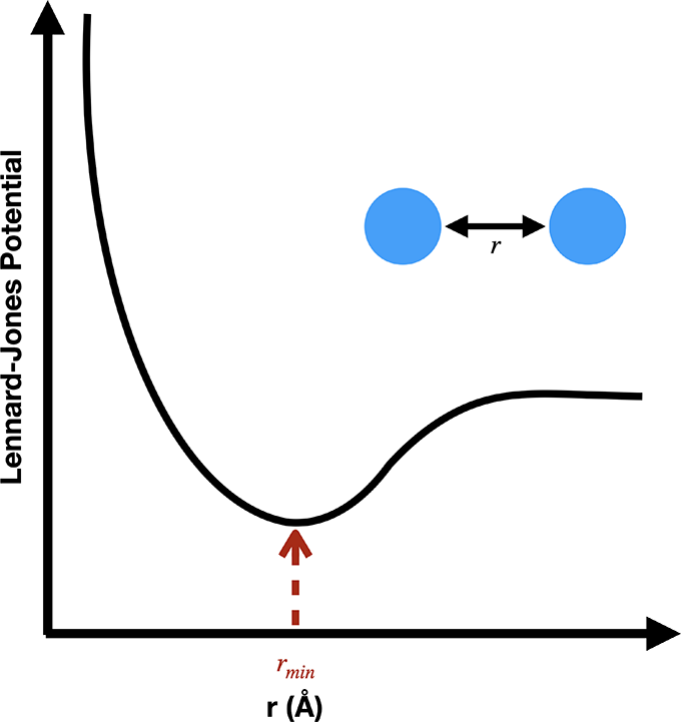
Lennard-Jones potential as a function of interatomic distance for a diatomic system.^22^

We can decompose the nonbonded potential energy function *U*_non*-*bonded_ as
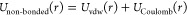
7which is a sum of the two primary types of interactions, van der Waals and Coulomb electrostatics.

van der Waals interactions (Figure 2) are modeled using a Lennard-Jones potential energy function and describe the competing attractive and repulsive forces between two atoms.These forces fall off quickly with distance so they are typically for atom pairs within a cutoff distance *r*_c_. This potential is defined as
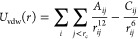
8where *A* and *C* are constants that depend on the types of atoms *i*, *j* involved in the interaction, *r*_*ij*_ is the distance between the atoms, *r*_c_ is the cutoff radius, and the notation *j* < *r*_c_ denotes the set of neighbors within the cutoff radius. This term is commonly truncated at approximately *r*_c_ = 10–14 Å.

The influence of the Coulomb electrostatic potential, in contrast to the Lennard-Jones potentials, falls off slowly with distance and is defined as
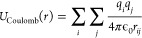
9where *q*_*i*_, *q*_*j*_ are the point charges for atoms *i* and *j*, *r*_*ij*_ is the distance between the atoms, and  denotes the Coulomb constant. Truncation of this term is difficult to achieve as atoms can have non-negligible interactions at arbitrarily long distances (see Section 3).

### 2.3 Integration Algorithms

Provided that forces have been computed, an integration algorithm is needed in order to drive forward the dynamics of the system.^14^ Two criteria used in the determination of the method are the approximate conservation of energy in the system and “time-reversibility”,^14^ which we will expand upon in Section 2.5. The Verlet algorithm^23^ achieves both of these criteria and is given by
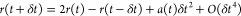
10where *O*(*δt*^4^) is the order of the local error of the calculation due to truncation of the Taylor series expansion about *r*(*t*). In order to compute quantities such as kinetic energy *K*, the velocities *v*(*t*) can be computed as
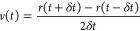
11

Alternative implementations of the Verlet method exist such as the “leap-frog” scheme that addresses issues around the handling of velocities that might introduce unnecessary error to the calculation. This “leap-frog” method directly computes *v*(*t*) at half time-step intervals and is given by
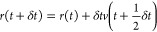
12
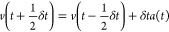
13

Lastly, we mention the velocity Verlet method as it is algebraically equivalent to the Verlet and leapfrog schemes, it is also implemented in most modern MD packages. The velocity Verlet integration scheme is given by
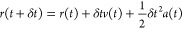
14
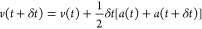
15

The integration methods covered here are not exhaustive, modern MD packages support additional algorithms for more sophisticated simulations.^24,25^

### 2.4 Role of Sampling in MD

A point that is often understated, especially when discussing MD with those who may not be domain experts, is that the exact motions observed of during an MD simulation are not meant to be taken literally.^14,26^ Another common question that frequently arises is how much simulation is required for an MD run. In either case it should be clarified that running a single simulation for an incredible amount of time does not guarantee that the biological event we are attempting to simulate even occurs, despite our knowledge about the time scales on which the event tends to occur.^27^ MD simulations are sensitive to their initial conditions and two nearby initial starting points will produce trajectories that diverge exponentially in time.^28^

When running simulations, we are concerned with the measurement of an observable *O* which depends on the microscopic system configuration, or *microstate*, *s*. For clarity, *s*_*i*_ = (*r*_*i*_, *p*_*i*_) where *r*_*i*_ is the position of the *i*^th^ atom and *p*_*i*_ is the momentum. The individual frames that are generated sequentially over the course of a simulation can be seen as a distribution of microstates^26,28^ in *phase space*. For simplicity, we use *s*(*t*) to refer to the microstate of the system at time *t*. It is possible to compute the value of *O* using the time average where all possible microstates will have been sampled:
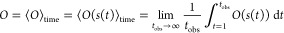
16

Clearly it is impossible to evaluate eq 16 as in reality we must choose a limit of time steps, *t*_obs_, as a computation budget we are willing to exert for an MD run. We now have a discrete form for eq 16:
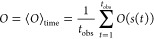
17however when computing *O*, it is important to understand whether enough exploration in terms of system configuration has been done rather than being purely concerned with the simulation time scale itself.

### 2.5 Relating Microscopic Behavior to Macroscopic Quantities by Statistical Mechanics

Statistical Mechanics provides a link between the behavior of a system at the microscopic level to its macroscopic properties we generally refer to as *O*, such as energy or entropy.^14,26,28^ We assume a probability density ρ(*s*, *t*) over the phase space that is governed by our choice of *ensemble*.^14,26,28^ The simplest ensemble is known as the *Microcanonical* or NVE ensemble which refers to constant moles (*N*), volume (*V*), and energy (*E*).^26,28^ We note here that the simulations covered in this work correspond to the NVE ensemble unless stated otherwise. The *Hamiltonian* governs the evolution of the system over time in phase space and represents the sum of the kinetic and potential energy of the system:
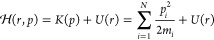
18where *N* is the number of atoms and *m*_*i*_ is the mass of the *i*^th^ atom.^14,26^ It can then be shown that Newton’s second law of motion can be derived from the Hamiltonian formulation by taking derivatives of ,
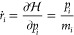
19
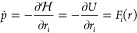
20then inserting back into eq 20 to yield Newton’s second law of motion:^26^
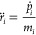
21
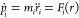
22

In subsection 2.3, we introduced the typical integration schemes used in MD, however we did not make the connection as to why the Verlet methods are preferred in our case of MD. Briefly stated, a numerical integrator that conserves  is known as a symplectic integrator.^14,26^ This property allows for the system dynamics to satisfy the conservation of energy requirement of the NVE ensemble.^26^ Furthermore, as Hamilton’s eqs 19 and 20 are symmetric in time, an integrator should be *time-reversible*.^14,26^

The density ρ(*s*, *t*) clearly depends on *t*, however as the system approaches equilibrium in the limit of time (that is, *t* → *∞*), then *∂ρ*/*∂t* = 0 and we arrive at ρ_eq_.^14^ Given the equilibrium density ρ_eq_ and the set of points in phase spaces for which ρ_eq_ is nonzero, the system is said to be *ergodic* if there exists at least one trajectory that, given sufficient time, visits all of the microstates in phase space.^14,26,28^ This is another way of saying that sampling a single system over infinite time is equivalent to sampling *s* over many systems frozen at a single point in time, provided the ergodic hypothesis holds.^14,26,28^ With ρ_eq_, we can then reformulate eq 17 by ignoring time and instead taking a weighted average of *O* according to the probability density given by ρ_eq_(*s*):
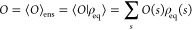
23

Thus, the predictive power of the MD method lies within the efficient and effective sampling of phase space, given the choice of ensemble.

## 3 Algorithms for Non-Bonded Force Computations

Computation of nonbonded interactions comprise the major bottleneck in MD simulations and according to Amdahl’s law should be the focus of acceleration efforts.^29^ Numerous algorithms have been proposed^30,31^ that are more efficient than the naive  direct computation with greater accuracy than a cutoff-based method.^31^ We discuss methods featured in the literature in the following subsections.

### 3.1 Particle Mesh Ewald

The Particle Mesh Ewald method, also known as PME, is a prominent algorithm for nonbonded electrostatic force calculations in biological simulations. There are a variety of PME formulations used in the literature.^32−34^ The common idea among these methods is the splitting of the slowly converging sum of electrostatic contributions in eq 9 into a short-range contribution, smooth long-range contribution and a “self” contribution. At a high level, the PME methods consists of five steps:^31^

1. **Charge assignment**: The charges in the simulation real space are “smeared” onto a uniform grid of points using a window function.
2. **Grid transformation**: The charge grid is then transformed from real-space to a reciprocal space by way of the Fast Fourier Transform (FFT).
3. **Multiplication by optimized influence function**: The components of the FFT-transformed charge grid are then multiplied by an optimal Green’s function. A Green’s function is a mathematical tool used to solve difficult instances of ODE’s and PDE’s.
4. **Transformation from reciprocal space back to real space**: An inverse FFT is performed to transform the perturbed charge grid from reciprocal space back to real space.
5. **Force assignment**: The forces from the charge grid are interpolated onto each atom in the simulation space using the same window function from step 1.

For a more in-depth description, we refer the reader to^30^ and to the relevant papers.^32−34^

### 3.2 Tree-Based Approaches

The Barnes-Hut algorithm^35^ and Fast Multipole Method (FMM)^36^ provide an efficient way of computing nonbonded electrostatic interactions by using an octree decomposition of the simulation space. The intuition behind these methods is that beyond a “well-separated” distance, the electrostatic contributions of these “far-field” particles to a reference particle become more similar to increasing distance. Interactions under this distance are interpreted as “near-field” and are much more sensitive to distance from the reference particle. The algorithms differ in the stopping criteria for the decomposition as well as the form of the potential function that aggregates the interactions of each “cell” in the octree. Whereas the Barnes-Hut method uses a simple sum aggregation of the cell, FMM uses “Multipole” expansion of the spherical harmonics of the cell, which gives a series of progressively finer angular features, that is “moments”: The order *p* of the multipole expansion are functions of an acceptable level of error ϵ where *p* is typically chosen as . The core of the Fast Multipole Method lies within a set of three translation operations that propagate the computed interaction information through the simulation tree data structure, an upward pass of information, and a downward pass stage as well as three translation operations; translation of a multipole expansion, conversion of a multipole expansion into a local expansion, and translation of a local expansion. For a more in-depth description of the tree-based methods, we refer the reader to the relevant papers.^31,35,36^

### 3.3 Multigrid and Multilevel Summation

Alternative hierarchical methods also exist, including the Multigrid^37,38^ and later Multilevel Summation methods,^39^ which exhibit linear asymptotic complexity in the number of atoms *n*. In contrast to the FMM and other tree-based methods, the Multigrid method hierarchically decomposes the simulation into a series of progressively finer potential grids rather than hierarchically decomposing the interaction pairs themselves.

### 3.4 Quality Measurements

Two metrics to assess the quality of a simulation are consistently mentioned in the literature; the Relative RMS Force Error and the Total Energy Fluctuation.^10,34,40,41^

The Relative RMS Force Error is used to validate a new design of an MD simulation which may potentially differ from a reference MD simulation in some way such as the precision used to compute forces, the cutoffs used for Lennard-Jones interactions, and so forth. The forces obtained from the high quality simulation are given for the *i*^th^ atom as , and the forces from the query simulation are given respectively as *F*_*i*_. Thus, the Relative RMS Force Error is defined as
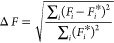
24

While Relative RMS Force Error can communicate how well a new simulation may reflect the behavior of a reliable reference point, it is difficult to determine whether the results of the simulation are physically plausible from this metric alone.

The Total Energy Fluctuation provides a measurement of physical plausibility by measuring the sum of the relative change in total energy, given by Δ*E*, of the physical system at each time step. The total energy *E* of the system is the sum of the kinetic and potential energy of the system:
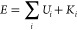
25where *U*_*i*_ is the potential energy for the *i*^th^ atom, and *K*_*i*_ is the kinetic energy for that atom. The kinetic energy for an atom in the simulation is computed as
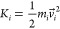
26where *m*_*i*_ is the mass of the *i*^th^ atom in the simulation and  is the atom’s velocity vector. The Total Energy Fluctuation is then defined as
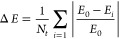
27where *E*_0_ is the initial total energy of the system and *E*_*i*_ is the total energy at time step *i*. In the ideal case, this value should approach 0, indicating that the total energy in the simulation has been conserved and the results are likely to be physically meaningful. In practice, an acceptable upper bound has been proposed as Δ*E* ≤ 0.003.^34^ However, the choice of this particular threshold for Δ*E* appears to be the product of a choice made for a specific simulation and does not necessarily apply to arbitrary simulation systems and or force fields.^42−48^

### 3.5 Comparison of Algorithms for Non-Bonded Interactions

Support for the Particle Mesh Ewald method is nearly ubiquitous among modern production MD software packages given that it is well studied, “easier” to implement efficiently compared to some tree-based approaches such as Fast Multipole Method (FMM),^36^ exhibits good asymptotic runtime in the number of atoms *n*, and is generally accepted to be “accurate” according to the metrics presented (eqs 24 and 27). Improvements have been made to the original PME method, such as the “Smooth” Particle Mesh (SPME) algorithm,^33^ which improves the accuracy of the interpolation from the simulation space to the charge grid that is input into the later Fast Fourier Transform calculation by way of B-spline interpolation. Additional variations of PME-based approaches include the *k*-GSE method, which uses a series of Gaussian kernels to perform the charge spreading step (Table 1).^34^

**Table 1:** Various Non-bonded Force Interaction Algorithms Featured in the Literature Covered in This Review

abbrev.	name
PME	Particle Mesh Ewald
SPME	Smooth Particle Mesh Ewald
*k*-GSE	*k* Gaussian Split Ewald
FMM	Fast Multipole Method
MGrid	Multigrid
BH	Barnes-Hut

## 4 GPU-Based Acceleration

### 4.1 GPU Architecture Overview

Graphics processing units (GPUs) have garnered much attention as accelerators for applications in machine learning and are noted generally for possessing the ability to exploit data parallelism inherent in certain classes of algorithms. Whereas a *Central Processing Unit* (that is, CPU) is designed to be capable of quickly switching between multiple serial tasks that may differ enormously in their control flow or required resources, GPUs are designed to trade-off the CPU’s cache and control resources per core for a greater number of lightweight compute cores.^49^

**Figure 3:**
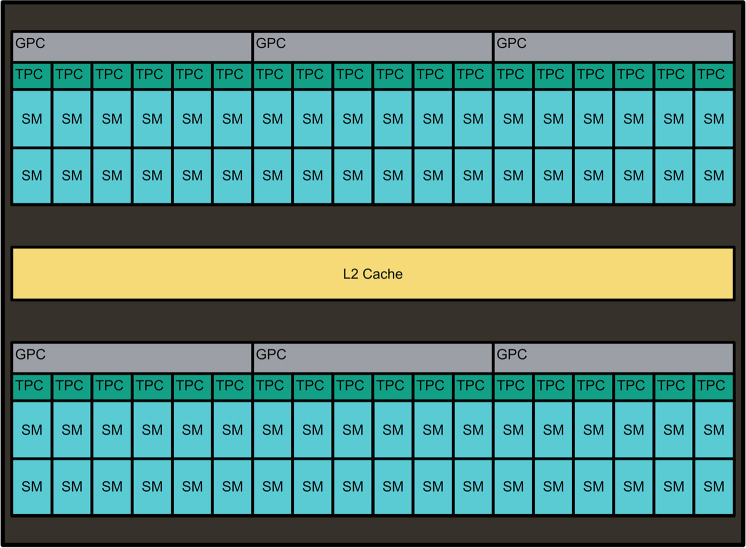
Description of an NVIDIA GPU architecture.^50^

**Figure 4:**
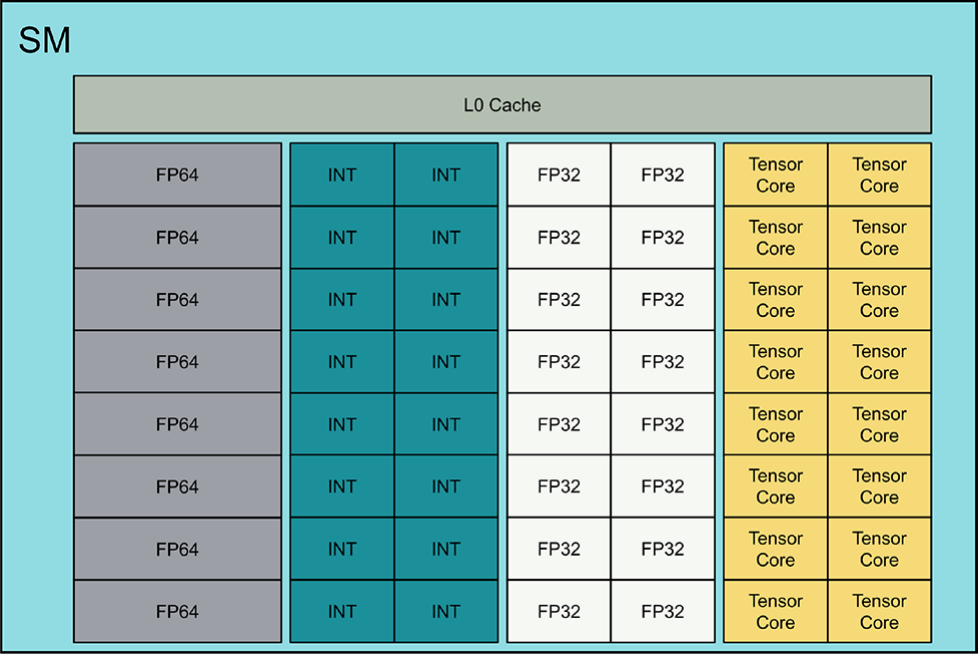
Description of an NVIDIA GPU Streaming Multiprocessor (SM) unit..^50^

In Figure 3, the components of the NVIDIA Tesla V100 GPU architecture are shown, indicating the GPU Processing Clusters (GPCs), each of which is composed of multiple Texture Processing Clusters (TPCs), composed of multiple Streaming Multiprocessors (SMs). The SMs, depicted in Figure 4, of the GPU are the fundamental computation unit and within these elements are special cores for floating-point and integer arithmetic as well as specialized tensor cores that accelerate matrix and tensor multiplications often encountered in deep neural networks. In order to exploit data parallelism, the V100 GPU utilizes a *Single Instruction, Multiple Thread* (SIMT) execution model, where a thread can be thought of as an arbitrary execution of the kernel.^51^ The SIMT execution model then is best thought of as an extension of *Single Instruction, Multiple Data* (SIMD) to multiple threads.^51^ For the sake of clarity, when referring to SIMD, we are referring to the array-processor defined in Flynn’s Taxonomy.^52^ In this model groups of threads are called *thread blocks*, which are decomposed into execution “warps”. Warp sizes are typically chosen as 32 across recent NVIDIA GPU architectures. Warps are then scheduled for execution across the SMs of the GPU, which can execute multiple warps in parallel among the SM compute cores. The hierarchy of SM groups in the GPU allow for the sharing of memory resources at various levels with L0/L1/L2 cache memories as well as a high bandwidth DRAM. Modern NVIDIA GPUs also feature NVLink interconnect technology, which allows for GPU-to-GPU data transfers at up to 160 Gigabytes/second, providing 5 times as much bidirectional bandwidth as PCIe Gen 3 x16.^53^

The advantage that GPUs provide relative to CPU implementations of MD simulations is precisely the SIMT model of parallelism. The number of threads that may operate in parallel are well into the thousands for modern GPUs, whereas the number of CPU threads on a consumer device remain limited to single digits. Even considering more advanced CPU architectures such as the IBM Power Series or special extensions to CPU instruction sets such as the Intel Advanced Vector Extensions^54^ which provide CPUs with enhancements for SIMD processing, GPUs remain far superior in exploiting data parallelism.^49^

In comparison to FPGAs, which can leverage the same data parallelism that makes GPUs favorable for MD, GPUs lack support for custom data-types that have been used in ASIC and FPGA architectures to maximize resource utilization. For example, ASIC and FPGA architectures allow for the designer to employ custom floating units that can be tuned to the most effective level of precision, while GPUs are limited to computing with 16-bit, 32-bit, and 64-bit floating point units that are included in the architecture. This flexibility in the representation of the data can allow for ASIC and FPGA designs to mix various levels of precision throughout the computation, for example to compute individual forces with 32-bit fixed point and then accumulate forces with 64-bit fixed point using dedicated hardware units (see corresponding discussion in Sections 5 and 6).

### 4.2 GPU MD Applications

Classical MD simulations were some of the earliest beneficiaries of GPU acceleration.^55,56^ This adoption was due in part to the availability of the CUDA GPU programming framework, drastically reducing the complexity of mapping a nongraphics application to the GPU.^55,57^ Additionally, GPUs were posed as solutions to the development of costly specialized processors due to their relatively low cost as a consequence of their popularity as accelerators for gaming.^55^ Subsequent years show an explosion of interest in the use of GPUs to accelerate Classical MD simulations, with increasing complexity of algorithms studied.^58^ A recent comprehensive review of the work in this field has been published^59^ and so we refer the reader there for more detail. In this overview, we focus primarily on the works published since.^59^

### 4.3 GPU Software Implementations

There exist numerous packages for MD itself, so we will not exhaustively study all those available in detail. We instead choose to highlight some of the popular packages and discuss their implementations comparatively.

NAMD (NAnoscale Molecular Dynamics)^60^ was the earliest adopter of GPUs among MD packages, leveraging the programmability provided by the Nvidia CUDA GPU programming library.^55^ NAMD is built using the C++ programming language and CHARMM++^61^ parallel computation library. The philosophy of NAMD is to make running simulations a simple process for the user, not to serve as a platform to be modified by the user extensively.^62^ NAMD is supported across Windows, OSX, and Linux operating systems. In the initial description of the GPU features for NAMD, the PME algorithm^55^ is presented as the kernel for nonbonded interactions. However, the description clarifies that the reciprocal space calculation for PME is actually implemented to run on the CPU, while a combination of short-range electrostatics and van der Waals interactions are the target for GPU acceleration. In addition to the reciprocal space calculation, all other steps (for example, bonded interactions, integration) of the MD simulation are carried out on the CPU. Subsequent releases of NAMD increasingly moved computation from the CPU to the GPU, notably all forces are now computed on the GPU including all computation related to the PME implementation (SPME).^62,63^ The latest version 3.0 of NAMD is being actively developed and represents a significant shift in the design philosophy.^62,64^ The NAMD developers note that the traditional decomposition of the MD algorithm across the CPU and GPU, where the GPU is tasked with force evaluations and the CPU with all other tasks related to integration, leaves higher-end GPUs idling for a significant portion of time.^62^ Currently, the developers of NAMD are developing a “GPU-resident” version where the MD computation is moved almost entirely from the CPU to the GPU to reduce costs associated with moving data between the CPU and GPU for each iteration of the MD simulation.^62^ A recent blog post by a collaborative team at Nvidia also details the most recent work in migrating the NAMD code from CPU to GPU.^64^ Preliminary results show a speedup of up to approximately 1.9 times faster than NAMD v. 2.13 when using the same GPU, the Nvidia V100.^64^ Lastly, it should be noted that current available version of the 3.0 alpha version of NAMD supports the GPU-resident approach for a single GPU at a time, with the focus being on being able to run more parallel instances of the same MD system, allowing for greater amount of sampling.^62^ The NAMD software is distributed free of charge with its source code.

GROMACS (GROningen MAchine for Chemical Simulation) is another popular open source MD package.^65^ The design philosophy of GROMACS is to run in as many different computing environments as possible, ranging from a laptop equipped with only a CPU to massive clusters of heterogeneous servers that may feature multiple GPUs per node. Version 4.5 of GROMACS was the first to feature GPU acceleration, with the entire MD calculation implemented on the GPU, similar to the current goal of the NAMD project we previously described.^66^ However, in contrast to the approach described by the NAMD authors, the latest iteration of GROMACS is targeting increasingly heterogeneous platforms, making use of both the CPU and the GPU implementation of the MD algorithm in what the authors refer to as a “bottom-up heterogeneous approach”.^67^ In order to accomplish this approach for acceleration of MD, GROMACS uses a scheduling protocol to allocate work between the CPU threads and GPU(s) efficiently, taking into account the particular topology of the compute environment as well as NUMA (Non-Uniform Memory Access) considerations when placing threads. The GROMACS software is able to assign force calculations to both the CPU and GPU to optimize communication and computation overlap, rather than explicitly placing all force calculations on a single device. Interestingly, this extends to the FFT calculations required for the PME algorithm; specifically, while the truncated real-space nonbonded interactions are carried out on the GPU, the CPU is used to compute the forces for the reciprocal space. Additionally, the GROMACS developers note their use of CUDA streams to allow for nonlocal nonbonded interactions to be sent to high priority queues across the various GPUs, allowing for preemption of the local kernel to return force calculations early. Additionally, GROMACS possesses the ability to offload entire MD iterations to the GPU. In order to minimize data transfer overheads, GROMACS uses the (relative to PCIe bandwidth with host CPU) higher bandwidth GPU-GPU interconnect (NVLink) to leverage data transfer capabilities between GPUs. The developers of GROMACS state further improvement to the load balancing capabilities for CPU and GPU task scheduling as well as continued work to overlap communication and computation as priorities. Recent work has extended GROMACS to use the FMM method, which the authors anticipate will become the algorithm of choice for “the largest parallel runs”.^67^ An implementation of FMM for GROMACS has since been published.^58^

Additionally, Amber is one of the most popular MD packages for Drug Design.^68^ The name Amber often refers to the set of force fields developed by the Amber project.^19,69,70^ Amber additionally provides extensive support for various types of simulations as well as analysis tools such as PTRAJ and CPPTRAJ.^71^ For the sake of our discussion, we are concerned with the GPU MD simulation program, *pmemd*.^72^ Version 11 of the Amber software was the first to provide support for GPU acceleration of the PME calculations. The most recent published description of the Amber software, corresponding to version 12, states that the entire MD calculation was moved onto the GPU.^72^ The authors note that their intention with version 12 of the software was to “port the exact equations as they are described in AMBER’s CPU code” to the GPU. A major point of emphasis was the introduction of a numerical precision model for the MD engine termed “SPFP” which combines single-precision floating point and 64-bit fixed point arithmetic.^73^ The distinction should be made that NVIDIA GPUs have not themselves supported fixed-point arithmetic at the hardware level so the implementation approximates this through software. As opposed to NAMD and GROMACS, the Amber implementation as presented in^72^ does not use table lookup to compute the forces between the atoms, choosing instead to directly evaluate the equations. The *pmemd* program supports running simulations using multiple GPUs by use of MPI. In the case of a multi-GPU run, all simulation data structures are replicated on each device to minimize data transfer overhead. The FFT calculation required for reciprocal space force evaluations in PME are implemented using the NVIDIA-developed cuFFT library.^74^ Additional features have been added to later iterations of Amber, as well as ongoing performance enhancements that are detailed in.^75^ Amber provides a number of free simulation and analysis tools under AmberTools, which is regularly updated. An extensive manual detailing the functionality available through the package is also regularly updated with each release. More advanced programs such as *pmemd* are available by way of a licensing mechanism.

### 4.4 Characterizing Performance of GPUs for MD

There is great interest currently in characterizing the performance of GPUs to better understand their scaling behavior. The Amber MD software package regularly publishes benchmarking data on their Web site.^76^

**Figure 5:**
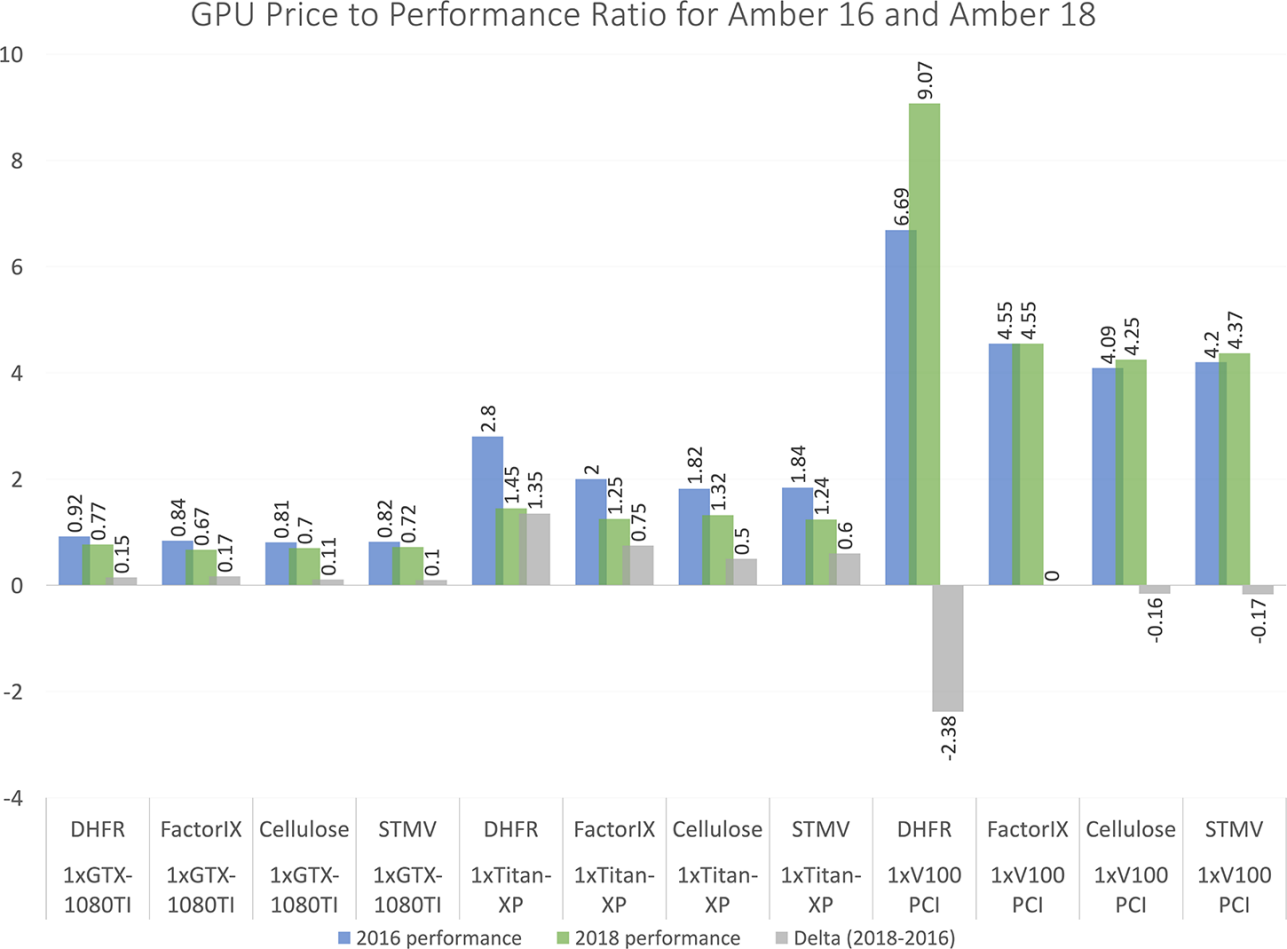
GPU price to performance comparison for Amber MD software for versions 2016 and 2018. The data are collected from the Amber Web site.^76^

In Figure 5, we summarize some of the recent analysis of price to performance ratios for several popular GPUs spanning from “consumer-grade” (NVIDIA GTX 1080TI) to “datacenter-grade” (NVIDIA V100) with respect to the Amber 2016 and Amber 2018 MD packages, a widely popular MD simulation package. The Amber software at each time point is examined across multiple commodity GPUs, and with each device a price-to-performance metric is reported, where the best values would be those approaching 0. In both cases, despite active development of the GPU implementation of the *pmemd* code,^75^ the price-to-performance exhibits a marginally decreasing relationship to theoretically more capable devices possessing progressively larger numbers of processing elements and larger global memories. Another observation is that the price-to-performance ratios for the top-end GPU, the NVIDIA V100, increases for all simulation types monitored in this study. It is not explicitly mentioned here why this is the case, analysis for other algorithms is also not included, and it is important to note that other MD software packages along with their specific algorithmic implementations may exhibit different behavior. As the more recent NVIDIA A100 begins to supplant the V100, based on the observations made here, extra performance will come at a premium that does not necessarily scale as favorably as it does for lower-end cards.

Recent benchmarking of the Gromacs^67^ software to characterize price-to-performance has also been performed.^77^ The main conclusions from this work agree with what has been observed for the Amber results; consumer grade GPUs provide the greatest price-to-performance as opposed to professional datacenter-grade GPUs. The conclusions of the work further imply that the growth of GPU capabilities or other unspecified architectures will lead to improved throughput of simulations in future works.

Recently, NAMD was used in a Gordon Bell prize winning work that investigated the use of large scale atomistic MD to illuminate mechanisms of SARS-CoV-2 spike dynamics.^78^ Two molecular simulations are studied in this case, an 8.5 million atom spike-ACE2 complex and a 305 million atom virion (that is, the complete virus structure). In the case of the smaller spike-ACE2 system, scaling to more GPUs quickly saturates performance, while for the larger virion system, the performance is nearly linear up to 512 compute nodes and then begins to saturate. The PME algorithm is used in both MD simulations, demonstrating the negative impact communication overheads have on simulation performance, resulting in marginally decreasing returns with each additional device added.

As stated previously in Section 4.2, the developers of NAMD are working to improve their implementation by offloading more routines from the CPU to the GPU, such as the numerical integration step. The analysis reported by the NAMD developers,^79^ shows the simulation throughput (measured in nanoseconds of simulation per day) of the NAMD 2.14 versus NAMD 3.0alpha9 versions running on an NVIDIA DGX-2 as a function of number of GPU devices. The projected performance assumes the ideal optimized version of the software with minimal dependency on the CPU, demonstrating an exponential growth in performance in this case. However, it is unclear at this time what the outcome of this optimization will ultimately achieve. The communication costs imposed with increased number of devices into the thousands will likely continue to limit performance of a GPU-centric implementation for acceleration of a single system, though these observations provide insight into the NAMD designers focus on the GPU-resident design which can be used to accelerate enhanced sampling algorithms.^62,80^

### 4.5 Discussion

GPUs have experienced enormous interest as general purpose accelerators given their low unit cost and increased accessibility through the NVIDIA CUDA, OpenCL, and AMD RocM libraries. Great interest remains in understanding the limits of the technology, evidenced across performance analyses for production MD software packages, such as Amber, Gromacs, and NAMD.^62,67,81,82^ Future work in improving the scaling of GPU implementations will pay particular attention to algorithms that address the efficiency of all-to-all communication patterns inherent to PME-based approaches.^58,67,83^ Integration with often GPU-centric deep learning tools will also play a significant role in addressing the efficiency of MD simulation.^78,84,85^

The primary advantage GPUs have exhibited to date compared to competing architectural solutions lie within the robust development resources available to researchers to map the nonbonded force calculations to the SIMT-paradigm GPUs exploit, coupled with their relatively low prices. It is also the case that more complex accurate algorithms, such as PME which depend on an underlying Fast Fourier Transform, have benefited from GPU acceleration. With tools such as the NVIDIA CUDA framework,^86^ GPUs are more favorable to alternatives such as FPGAs for the relative ease of development. With that said, GPUs lack hardware flexibility that can allow the programmer to define custom data-types that may make better use of the chip resources or allow specific calculations to be done more efficiently over the course of the 10^9^–10^12^ iterations required.^7^ GPUs are also defined as general purpose processors and so while instructions can be carried out in parallel on separate data, there remains latency in execution due to control flow overhead such as process synchronization and instruction fetching. Imbalances in computational capacity of the GPU versus the bandwidth for which data can be supplied result in challenges to keep the processors effectively occupied. FPGAs in contrast leverage a spatial architecture in which the data flows through a user defined pipeline that alleviates the aforementioned sources of control flow latency and are also directly equipped with high bandwidth I/O interfaces^49,87^ (see the corresponding discussion in Section 5).

## 5 Reconfigurable Architectures for MD

### 5.1 Overview of FPGA Architecture

**Figure 6:**
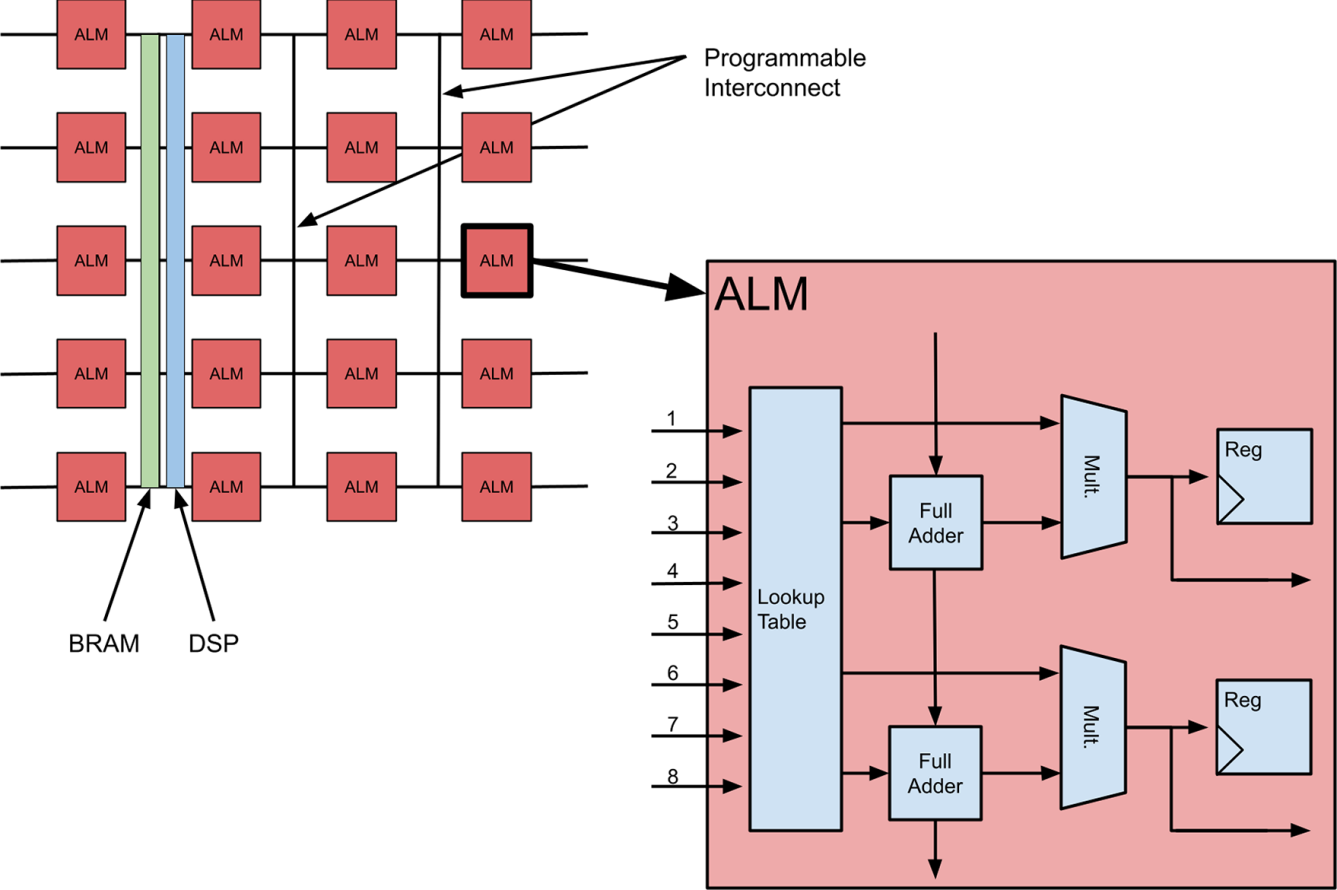
Description of an FPGA architecture.

At a high level, an FPGA can be described as an array of programmable logic blocks and programmable interconnect between those blocks that can be configured after fabrication to implement arbitrary program logic.^88^ An example of an Intel FPGA (formerly Altera) architecture is shown in Figure 6.The Adaptive Logic Modules (ALMs) are the fundamental compute units of an FPGA. ALMs consist of a *Lookup table* (LUT) which is a memory that maps address signals as inputs and the outputs are stored in the corresponding memory entries.^88^ LUTs can be programmed to compute any *n*-input Boolean function. *Full Adders* perform addition and subtraction of binary inputs. *Flip Flops* (FF) are the basic memory element for the FPGA and serve as registers allowing the ALM to maintain state. The FPGA architecture then interconnects many of the ALMs through programmable interconnections forming an array of processing elements.^88^ Coupled with this mesh of ALMs are specialized or “hardened” elements such as on-chip Block RAM (BRAM) and Digital Signal Processing (DSP) blocks.^88^ BRAMs are configurable random access memory modules that can support different memory layouts and interfaces.^88^ DSP blocks help compute variable precision fixed-point and floating-point operations.^49^ Additionally, modern FPGAs also include microprocessors that serve as a controller, allowing a user to run an operating system such as Linux in order to leverage facilities such as the device’s communication drivers or running high-level programming languages such as Python.^88^ Lastly, FPGAs use I/O blocks that allow for direct low-latency interaction with various network, memory, and custom interfaces and protocols.^49,88^

FPGAs, unlike GPUs and CPUs, are a *spatial* architecture, in that the hardware directly and continuously executes a spatial hardware circuit representation of the software. This eliminates control flow overhead that is encountered in general purpose architectures which require instruction-fetch as well as process scheduling and synchronization which can be detrimental to performance.^87^ The programmable hardware approach instead allows the data to flow through the pipelines that the designer specifies in software or through Computer Aided Design (CAD) tools.^49^ In contrast to ASIC architectures, which are not able to be updated once the design has been fabricated, FPGAs are able to be reconfigured with updates to the algorithms as advances are made.

Despite the ability to be reconfigured with arbitrary logic, updating an algorithm design for an FPGA is a significantly slower process than it is for CPUs and GPUs, with compilation time scales varying between minutes to a few days.^89^ The learning curve for designing FPGA implementations has historically been much steeper than for CPUs and GPUs due to a dearth in higher level development tools.^89^ However, as industry trends suggest heterogeneous architectures as a solution for the issues faced by general purpose processors in the trade-offs required for power versus efficiency,^49,90^ a number of tools have been developed to ease the development of programs that exploit the FPGA in anticipation of a heterogeneous processing future.^91−97^

While early FPGA-based MD implementations were constrained to use vendor-specific toolchains that required VHDL or C-like dialects (SRC MAP C) to program, modern tools include Intel Quartus Prime, Xilinx Vivado, and OpenCL toolchains, which allow for a higher-level C/C++ interface to implement designs. In Table 2, we document the FPGA hardware utilized in MD simulations reported in the literature. The number of ALMs, embedded memories, dedicated multiplier blocks, and transceiver I/O rates have all increased by several orders of magnitude from the initial period of work. In Table 3, we provide a summary of the development tools used in the implementations reported in the literature. Recently, more attention has been paid to FPGAs for MD due to advances in the hardware itself amortizing the relatively higher cost as compared to GPU accelerators and thus addressing a major issue with their viability.^98,99^

**Table 2:** Characteristics of FPGAs Featured in Molecular Dynamics Simulations

ref	accelerator	ALMs	I/O band	embed. mem.	mult. blocks
(98, 99)	Intel Stratix 10	933 120	28.3 gb/s	253 Mbits	11 520
(100)^a^	Xilinx V5 LX 330T	51 840	3.75 gb/s	11 664 kbits	192
(29, 101, 102)	Xilinx XC2VP100	99 216	3.125 gb/s	7992 kbits	444
(103, 104)	Xilinx XC2VP70–5	74 448	3.125 gb/s	5904 kbits	328
(29, 102, 105−108)	Xilinx XC2 V6000	33 792	840 mb/s	2592 kbits	144
(109)	Xilinx Virtex-E 2000E	43 200	622 mb/s	655.36 kbits	0

a Denotes FPGA was used for simulation of performance.

**Table 3:** Description of the Development Environments Featured in the FPGA-Based MD Simulation Literature

ref	year	dev. board	dev. framework
(109)	2004	TM-3^110^	VHDL
(103, 111)	2005	WildstarII-Pro^112^	VHDL
(105)	2006	SRC 6 MAPstation	Carte
(102)	2006	SRC 6 MAPstation (E/C)	Carte
(106, 107)	2006	SRC 6 MAPstation (E)	Carte
(104)	2006	WildstarII-Pro^112^	VHDL
(101)	2007	SRC 6 MAPstation (E)	Carte
(29)	2008	SRC 6 MAPstation (E/C)	Carte
(113)	2011	Gidel PROCStar III	Proc Dev. Kit
(98, 99)	2019	Intel Stratix 10	Intel Quartus Prime Pro

### 5.2 Early FPGA Work

One of the earliest examples of applying FPGA accelerators to the problem of molecular dynamics is ref (109). The motivation of the work emphasized the power of reconfigurable processors to address the bottlenecks posed by the high costs of ASICs and the expensive memory hierarchies in general purpose processors. This work focuses solely on the short-range Lennard-Jones interactions while ignoring the Couloumb electrostatics to simplify the implementation.

The system consists of the PairGen, which generates the neighbor lists for each atom in the simulation; the Force computer, which computes the forces for the atoms using table look ups to retrieve precomputed parameters; and the acceleration update, which then is forwarded to the Verlet integration update which writes the new atom positions to the onboard memory. Fixed-point representations are used exclusively, each tuned specifically to the dynamic range of each quantity using a two particle system at various distances to precompute their possible values in order to minimize resource consumption. The design was implemented in VHDL. The simulation used for the analysis consisted of 8192 atoms. The FPGA system as configured gives a per-time step performance of 37 s, while the general processor benchmark (Intel Pentium 4 2.4 GHz, single core) is able to achieve 10.8 s. The authors claimed that with improvements to the memory systems as well as migrating to more up to date FPGA hardware with a higher clock rate would improve the speedup from 5.1× to 21×.

The work of ref (103) improves upon the shortcomings of ref (109) with an FPGA architecture that includes both Coulomb and Lennard-Jones forces. The design was implemented on a single Xilinx Virtex-II-Pro XC2VP70 FPGA and used fixed-point arithmetic throughout the implementation, and the force computations were carried out using lookup tables with interpolating polynomials, similar to ref (109). Furthermore, the work improves upon ref (109) by reporting a 57× speedup with respect to a single-core Intel Xeon 2.4 GHz CPU, with a benchmark similar to the previous work. Considering that most of the implementation was similar, the starkest difference was that the FPGA used in ref (103) had nearly twice as many ALMs as those used in ref (109) (74 448 vs 43 200), higher transceiver I/O bandwidth (3.125 gb/s vs 622 mb/s), more embedded memory (5904 kbits vs 655.36 kbits), and dedicated multiplier blocks (328 vs 0). Therefore, by using more capable hardware with a similar force calculation allowed the accelerator to vastly outperform the initial FPGA designs.

### 5.3 Toward Production FPGA-Based MD

While the initial studies of refs (103, 108, and 109) focused on proof-of-concept implementations, ref (102) investigates how one could go about porting a “production” MD system to an FPGA architecture, citing issues with ref (109) and^103^ being their focus on a “textbook” data set and the use of simplistic nonbonded force algorithms. Another point of criticism with the previous work was the use of “textbook” MD codes as general processor benchmarks, therefore the NAMD MD software^62^ is compared with the FPGA results and serves as the starting point for the FPGA implementation. The authors document their design process from extracting the relevant kernels from the NAMD code, implementation on the FPGA using MAP C, optimization of the initial designs using multiple target platforms of the SRC MAPStation, comparing the two featuring the Xilinx XC2 V6000 and Xilinx Virtex-II Pro XC2VP100 FPGAs. Their FPGA implementation is said to closely follow the original implementation (in Charm + +) including the use of SPME for nonbonded force calculations, improving upon the brute-force approaches of refs (103 and 109) Despite the increased design complexity, the work reports a 3× speedup versus the CPU baseline (dual-core Intel Xeon 2.8 GHz) using a data set of 92 224 atoms.

The work of ref (104) also proposes to address some of the issues with the earlier work by expanding the number of particle types, demonstration of integration with existing software (that is, ProtoMol^114^) similarly to,^102^ and general architectural improvements such as the introduction of “semi-floating point” representations for force calculations and optimizations to reduce the size of the lookup tables. The improved design yields a stable simulation of the bovine pancreatic trypsin inhibitor (BPTI) when considering total energy fluctuation (approximately 0.014 J) relative to the CPU benchmark. However, performance metrics are reported using the “textbook” data set of 8192 atoms and use the direct  nonbonded force calculation for the most competitive case, reporting a 15.7× speedup over the CPU benchmark (dual-core Intel Xeon 2.8 GHz).

A later paper^100^ improves upon ref (104) by implementing a more sophisticated algorithm (Multigrid^37^) to compute the short-range Lennard-Jones and Coulomb forces. The updated design is then compared against ProtoMol^114^ and NAMD CPU benchmarks. The FPGA design presented in ref (100) is validated using a 10 000 time step simulation where total energy fluctuation is measured. Compared to the results collected from a ProtoMol CPU implementation (dual-core Intel Xeon 2.8 GHz), a similar energy fluctuation is reported for both the FPGA design^100^ and ProtoMol.

The work of ref (105) claimed to be the first FPGA implementation of a realistic nonbonded force calculation algorithm in an FPGA-centric hardware acceleration platform, in contrast to the previous work of ref (109) (Lennard-Jones only),^103^ (simple data set). For the sake of brevity, the culmination of these works is presented in ref (29), which also provides the first study of parallelization of FPGA-based MD in a cluster of “reconfigurable-hardware-accelerated nodes”. The results of the scaling analysis were somewhat disappointing. On the one hand, as the number of nodes increases (using 1 Xilinx Virtex-II XC2 V6000 FPGAs per node), the latency per MD time step decreases. On the other hand, the decrease in latency compared to the CPU benchmark (dual-core Intel Xeon 2.8 GHz) is significantly worse than the CPU benchmark as the speedup from using 1 node to using 8 nodes drops from 2.08× to 1.51 ×. It is worth noting that the authors chose to implement their own software for the MD simulations for the CPU-based and FPGA-based implementations, therefore it is difficult to directly compare to other works while also considering this was the first scaling analysis done for FPGA-based MD.

The work of refs (101 and 115) demonstrates a design that makes use of the two Xilinx XC2VP100 FPGAs available on the SRC Series E MAPstation. Effectively, one of the FPGAs is used as a controller for the nonbonded force calculations, handling memory accesses from the host machine and running the outer loop of the PME calculations which are pipelined between both FPGAs. The second on-board FPGA is exclusively dedicated to the inner loop, the force calculations for a single atom and its “neighbors”. A major focus of this study was to present a highly optimized FPGA-based MD accelerator with what was at the time of publication, outdated resources thus suggesting the improvements in performance shown here (approximately 3×) to upward of 12× to 15×.

### 5.4 Recent Developments in FGPA-Based MD

The first end-to-end FPGA implementations of MD are presented in refs (98 and 99). The authors first develop an implementation that includes only the direct-evaluation component of the PME algorithm in ref (99) and then go on to include the full PME calculation and its FFT kernel dependencies in ref (98).

One of the primary contributions of both works^98,99^ is a detailed study of the trade-offs of various implementation designs. Of particular interest are the memory mapping schemes of the simulation “cells”, the distribution of work for the force calculations, and various implementations of the nonbonded force pipelines. For the *Dihydrofolate Reductase* (DFHR) test case of 23 558 atoms, the best design uses separate memory modules for each simulation “cell” along with a work distribution in which each pipeline works on its own “homecell”. The FPGA implementation achieves an approximately 10% speedup over the best GPU (NVIDIA Titan RTX) performance. Additionally, the FPGA implementation using first-order interpolation is shown to have a similar energy conservation as the Amber CPU benchmark. However, when the authors extend their analysis to include molecular systems that vary in their size and density, there is no design that consistently outperforms the others across all cases.

### 5.5 Discussion

A quantitative as well as qualitative summary of the work in FPGA-based acceleration for which a simulation was demonstrated is provided in Tables 4, 5, and 6.

**Table 4:** Quantitative Characteristics of Simulations Approached with FPGA-Based Acceleration

ref	year	#atoms	force prec.	box-size
(109)	2004	8192	50-bit Fixed	not reported
(103)	2005	8192	48-bit Fixed	not reported
(111)	2005	8192	35-bit Fixed	not reported
(105)	2006	32 932	17-bit Fixed	73.8 × 71.8 × 76.8 Å^3^
(102)	2006	92 224	32-bit Float	108 × 108 × 72 Å^3^
(106, 107)	2006	32 932	32-bit Float	73.8 × 71.8 × 76.8 Å^3^
(104)	2006	8192	35-bit Float	64 × 50 × 50 Å^3^
(101)	2007	23 558	32-bit Float	62.23 × 62.23 × 62.23 Å^3^
(29)	2008	32 932	32-bit Float	73.8 × 71.8 × 76.8 Å^3^
(99)	2019	20 000	32-bit Float	59.5 × 51 × 51 Å^3^
(99)	2019	20 000	32-bit Float	59.5 × 51 × 51 Å^3^
(98)	2019	23 558	32-bit Float	62.23 × 62.23 × 62.23 Å^3^
(98)	2019	23 558	32-bit Float	62.23 × 62.23 × 62.23 Å^3^

**Table 5:** Quantitative Characteristics of FPGA-Based Simulation Performance

ref	year	time/day	speedup	benchmark
(109)	2004	2.34 ps	0.29×	Intel Pentium 4 2.4 GHz
(103)	2005	517.4 ps	57×	Intel Xeon 2.4 GHz
(111)	2005	3.8 ps	51×	Intel Xeon 2.4 GHz
(105)	2006	0.188 ns	2.72×	Intel Xeon 2.8 GHz
(102)	2006	28 ps	3×	Intel Xeon 2.8 GHz
(106, 107)	2006	0.22 ns	1.9×	Intel Xeon 2.8 GHz
(104)	2006	0.72 ns	15.7×	Intel Xeon 2.8 GHz
(101)	2007	0.2 ns	3.19×	Intel Xeon 2.8 GHz
(29)	2008	246.86 ps	2.08×	Intel Xeon 2.8 GHz (dual-core)
(99)	2019	1.4 μs	96.5×	Intel Xeon
(99)	2019	1.4 μs	3.29×	NVIDIA GTX 1080Ti
(98)	2019	630.25 ns	25.3×	Intel Xeon
(98)	2019	630.25 ns	1.1×	NVIDIA GTX 1080Ti

**Table 6:** Qualitative Characteristics of Simulations Approached with FPGA-Based Acceleration

ref	year	force	LJ	coul.	alg.	arch.
(109)	2004	LUT	yes	no	direct	full MD
(103, 111)	2005	LUT	yes	yes	direct	nonbond only
(105)	2006	LUT	yes	yes	SPME	nonbond only
(102)	2006	LUT	yes	yes	SPME	nonbond only
(106, 107)	2006	direct	yes	yes	SPME	nonbond only
(104)	2006	LUT	yes	yes	direct	nonbond only
(101)	2007	direct	yes	yes	PME	nonbond only
(29)	2008	direct	yes	yes	SPME	nonbond only
(113)	2011	direct	yes	yes	PME	nonbond only
(99)	2019	LUT	yes	no	direct	full MD
(98)	2019	LUT	yes	yes	PME	full MD

In terms of throughput, measured as nanoseconds per day, time scales achieved by FPGA-based designs have improved by over 6 orders of magnitude.^98,99,109^ It is important to note however that much of the work reported for FPGA-based MD were not evaluated using production-level software on either the CPU or for the FPGA. Exceptions to this are the work of ref (102) which closely followed the NAMD^62^ source code and^101^ which closely followed the Amber^81^ source code. In terms of the general processor benchmarks, the reported relative speedup versus CPUs has consistently outperformed, though not by a large margin. Considering the throughput performance versus GPUs first reported in refs (98 and 99), while there is evidence of an improvement, it remains modest.

FPGA-based implementations have lacked evaluation in the production setting, considering the majority of the work has been relegated to standard benchmark data sets. Therefore, there has not been an exceptionally large simulation considered using FPGA hardware in the number of atoms or the size of the simulation box.

As work in the field progressed with hardware advances, designs have eschewed Fixed-point arithmetic for Single precision Floating Point in order to target an appropriate level of accuracy for production MD. While it is difficult to estimate the minimum precision required for all simulations, the work of refs (98, 99, and 113) provide the most comprehensive analysis of this. The most current work^98,99^ demonstrates that in their design, the use of Single Floating-point versus 32-bit Fixed point is more efficient in terms of ALMs and BRAM utilization. The main bottleneck presented by FPGA hardware has instead been resource utilization for force calculation pipelines. As the hardware has progressed, the number of pipelines working in parallel has increased several orders of magnitude.^99,102^

The vast majority of the FPGA-based designs have served as accelerators connected to a host machine to compute pairwise interactions given a cutoff threshold. Considering that the outer loop of the simulation was generally run using a CPU based code for the more complicated nonbonded interaction algorithms such as PME, this paradigm presented a clear bottleneck for memory accesses. The work of ref (29) addressed this in their design by utilizing the on-chip BRAM to store intermediate force calculations before making updates to the on board memory. Even with designs to optimize memory requirements,^29,101,102^ it was not feasible to remove the host bottleneck until recent work demonstrated fully end-to-end simulations completely on FPGAs.^98,99^ Additionally, the work of refs (98 and 99) provided additional investigation into the use of direct force calculations versus the use of look-up tables, deciding to use lookup tables to conserve DSP utilization with minimal loss in accuracy.

Lastly, as the applications have progressed, as have the available development tools. The early work in FPGA-based MD has either made use of VHDL^103,104,109,111^ or vendor-specific toolchains featuring C or FORTRAN interfaces, namely Carte and Gidel Pro Dev. Kit.^101,102,105−107,113^ The complexity of development on FPGAs versus other mainstream computing architectures such as CPUs or GPUs has been the primary hurdle to their adaption in a broad class of applications.^89,116^ More recently, additional tools such as Xilinx Vivado, OpenCL, and Intel Quartus Prime have provided developers with a richer set of tools with C/C++ interfaces to FPGA-based designs. Additional work in scaling FPGA implementations to multiple devices is ongoing. Industry trends suggest that in the near future heterogeneous architectures will become the norm, along with software stacks to ease the deployment of software in these environments.^49^

## 6 ASIC-Based Acceleration

Application Specific Integrated Circuits, or ASICs, are specialized hardware that can be tuned to solve specific problems very efficiently. In the context of MD simulations, most of the early work, as well as the most important advances, have been as a result of ASIC designs.^117−120^

### 6.1 Early ASIC Development

One of the earliest examples of an MD specific ASIC appeared in 1982^121,122^ for a condensed matter physics application. At the time of this work, MD simulations were typically constrained to be on the order of a few hundred atoms with the largest only approaching the order of a few thousands of atoms on modest time scales. The Delft Molecular Dynamics Processor (DMDP)^121,122^ was designed solely to accelerate the nonbonded force calculations with the host computer tasked with all other steps of the simulation. By keeping all particle data in the DMDP’s local memory and using fixed-point arithmetic (24 bits for position data, 32 bits for velocity data), the DMDP was able to achieve performance comparable to a Cray-1 supercomputer^123^ at reportedly less than 1% of the cost.

### 6.2 GRAPE (GRAvity PipE) and MDGRAPE

The Riken Institute-developed GRAPE/MD-GRAPE architectures for various instances of the *n*-body problem have led to numerous Gordon-Bell prizes over the years.^124^ The first GRAPE VLSI chip was introduced in 1990^125,126^ reporting performance comparable to the CRAY-XMP/1 supercomputer at a cost reportedly 10 000× less. GRAPE was proposed as an alternative to more complicated algorithms exhibiting favorable asymptotic complexity, by instead choosing the brute-force calculation of the direct interactions between particles with the use of a cutoff. The proposed design^125^ was similar to that of the DMDP with a host compute node managing the overall simulation with the GRAPE chip exclusively computing the long-range interactions.

While the early demonstrations of GRAPE were primarily focused on celestial systems simulations,^126^ the authors emphasized the flexibility of the machine to potentially handle arbitrary instances of the *n*-body problem ranging from the simulation of galaxies to that of biological macro-molecules such as proteins by “simply” replacing the potential energy function. Notably, the first GRAPE was apparently lacking this feature as it was not until several years later that the MD-GRAPE ASIC was introduced^127−129^ as an extended version of GRAPE that could support an arbitrary potential. The hardware architecture of MD-GRAPE was similar to that of previous ASICs^121,122^ in that the ASIC would be tasked with performing only the direct force calculations.

Work on improving the MD-GRAPE system continued for some time afterward with MDGRAPE-2^130^ increasing the number of pipelines in the chip (from 1 to 4), clock frequency, use of double floating point precision instead of 80-bit fixed point, and support for Direct Memory Accesses (DMA), while achieving a sustained performance of 15 billion floating point operations per second (that is, gigaFLOPs or GFLOPS) which was equivalent to the peak performance of other top high-performance computers at the time. Additionally, the overall workflow supported more advanced Ewald-based nonbonded forces. However, MDGRAPE-2 was restricted to “real-space” accelerations. Functionality for computing Fast Fourier transforms was implemented instead as a separate accelerator, “WINE-2”.^131,132^

The subsequent MDGRAPE-3 chip^118^ was reported as having a particularly high development cost at approximately USD 20 million. The authors emphasize that at less than $10 USD per GFLOP, the system was cost-effective unlike previous works^122^ or other notable high-performance general purpose computers such as the IBM BlueGene/L^132^ (approximately $140 USD per GFLOP). A number of molecular dynamics packages including Amber (version 6)^68^ and CHARMM (Chemistry at HARvard Macromolecular Mechanics)^133^ were already ported for MDGRAPE-2 and were also compatible with the MDGRAPE-3 chip.

While the MDGRAPE-3 system was demonstrated as the first quadrillion floating point operations per second (that is, petaFLOPS or PFLOPS) machine, significant bottlenecks remained in its design an accelerator attached to I/O buses of a host. MDGRAPE-4^134^ integrates the functions of both a host and the accelerator as a system on a chip (SoC), removing host communication bottlenecks. The MDGRAPE-4 chip^134^ consists of dedicated hardware for force pipelines, general processor cores along with a control general processor, network interfaces units, as well as an FPGA interface and memory units. The general processing cores (GP) are grouped onto each board in 8 blocks, and each general processor has 8 cores. The control general processor (CGP) is situated in the middle of the board and controls the execution flow of the force calculations by communicating with each of the general processors, the force pipelines (PP), and the network interfaces (NIF). Whereas the force evaluation pipeline spanned 80% of the die areas in MDGRAPE-3, it is reduced to only 20% by comparison in the MDGRAPE-4.

Force calculations are computed using mixed precision and formats. Input coordinates are expressed in a 32-bit fixed-point format as the dynamic range is expected to be relatively “small”. Force computations are expressed with a single-precision IEEE-754 floating point. Force summation is done using 32-bit fixed point. MDGRAPE-4 additionally differs from MDGRAPE-3 by calculating Couloumb and van der Waals forces and potentials simultaneously, whereas MDGRAPE-3 can only compute one at a time. Further, MDGRAPE-4 exploits Newton’s third law to reduce the computational cost by half. MDGRAPE-4 has 64 pipelines in eight blocks on each board, can handle 51.2 × 10^9^ interactions per second, with peak system performance equivalent to 2.5 trillion floating point operations per second (that is, teraFLOPS or TFLOPS). MDGRAPE-4 uses the Gaussian Split Ewald method for force calculation^34^ as it is quoted as possessing a good property for acceleration by specialized pipeline due to the Gaussian spread function having a spherical symmetry. The MDGRAPE-4 is programmed using C and assembly language. The authors in ref (134) ported the GROMACS^66^ “mdrun” engine to their architecture.

They also leave open in their discussion of what the optimal configuration for global memory should be and point to the fact that strong scaling of MD simulations is becoming more difficult and requires increasing amounts of specialization to improve performance, it may be worthwhile to investigate ways to improve cost/performance. They also point out that systems with a single chip or on a single module will be solutions that could address the cost/compute power concerns.

MDGRAPE-4a was subsequently developed to improve data and message flow efficiency and was applied to simulating potential therapeutics for the SARS-CoV-2 virus.^135,136^ The developers achieved performance of roughly 1 μs per day for the systems including about 100 thousand atoms using timesteps of 2.5 fs. Despite the improvement however, the authors still point out that the most recent Anton system is estimated to be 1–2 orders of magnitude ahead of the MDGRAPE-4a machine.^136^ The full MDGRAPE-4a system cost approximately $6.5 million USD to develop, which is significantly less expensive than that of MDGRAPE-3^118^ which was reported cost approximately $20 million USD but remains well beyond the reach of many researchers in terms of available development resources to replicate a design.

### 6.3 Anton

Unlike the first iterations of GRAPE/MDGRAPE, the principal task the Anton^117^ machine was designed for was to accelerate “very-long” time scale trajectories of biological entities such as proteins. In contrast to the MDGRAPE-3 architecture which split the MD tasks between a host microprocessor and a number of accelerator chips, the Anton ASIC utilized a system-on-a-chip SoC design with dedicated hardware to perform all tasks on chip or in a network of chips.

The Anton ASIC consists of 4 subsystems. The high-throughput interaction subsystem (HTIS)^137^ computes electrostatic and van der Waals interactions. The flexible subsystem^138^ controls the ASIC while handling tasks such as bonded force calculations, FFT, and integration. The communication subsystem handles communication on chip and between chips with 5.3GB/s communication bandwidth between chips. The memory subsystem provides access to the attached DRAM while also providing special memory write operations that support accumulations of force, energy, potential, and charge spreading. The HTIS is programmable by the use of SRAM lookup tables to allow for changes in force fields in ongoing research. It is not clear however to what extent this module can be modified to handle the recent work in force field development, an active area of research in biochemistry.

At the heart of the HTIS are the pairwise point interaction modules (PPIMs) and their pairwise point interaction pipelines (PPIPs). Anton distributes particle interactions using an efficient method that minimizes communication costs associated with importing and exporting atomic information among the nodes while also achieving scaling benefits with increasing number of nodes.^34,139^ The particles assigned to each node are referred to in two separate sets; the set of *tower* particles and the set of *plate* particles. The determination of a tower versus plate atom is based upon a set of criteria that is specified in detail elsewhere.^34^ The PPIM is distributed to a set of *tower* particles *T* and a set of *plate* particles *P*, for which the Cartesian product of the two sets is computed to form the set of possible pairwise interactions. Using a set of multiple *match units* in parallel, the PPIM filters the particle pairs for criteria assessing their suitability for an interaction evaluation. After filtering, the PPIPs compute the interactions between the particle pairs. The PPIPs feature optimized numerical precision for each functional unit along the datapath, maximizing the use of die area. The HTIS then accumulates the results and writes the results to the memory subsystem.

In terms of hardware capabilities, the subsequent Anton 2 ASIC^140^ improves upon the original in nearly every conceivable way. The Anton 2 chip is fabricated using 40 nm process technology versus 90 nm for the original. The HTIS supports a maximum of 32 768 atoms, an increase from 6144. The data bandwidth for the torus neighbors has also been increased from 221 Gb/s to 1075 Gb/s. A priority of this updated design was to increase the overlap of communication with computation in order to make more effective utilization of the hardware capabilities. In addition to hardware improvements, all embedded software on Anton 2 is written using C++ using a ported version of the GCC compiler. This is an improvement upon the mix of C and assembly required for the original Anton.

The latest iteration of the Anton series was recently introduced as Anton 3.^139^ Anton 3 includes improvements to the physical capabilities of the hardware from Anton 2, featuring 1500× increase in transistor count, 107× increase in SERDES Bandwidth (GB/s), 1290× increase in the number of PPIP modules, as well as 264 newly developed specialized bond interaction calculators. Anton 3 also features several improvements in the PPIP modules which feature a special bonded interaction calculator that makes for a higher throughput calculation while requiring one-third the die area of a geometry core which was previously used to compute bonded forces. For nonbonded forces, Anton 3 introduces two specialized PPIP modules, the Big PPIP and the Small PPIP. The Small PPIP module computes interactions with *r* > 5°A with specialized simpler logic at lower precision (14-bit datapaths) allowing for three Small PPIPs to fit in the area as one Big PPIP. Conversely, the Big PPIP handle interactions with *r* ≤ 5°A at increased precision (23-bit datapaths). Other significant improvements to the filtering and communication protocols for mapping interactions to PPIPs achieve improved load balancing due to the novel *Manhattan Method* for distributing the computation of nonbonded interactions, which is introduced in the work describing Anton 3.^139^

### 6.4 Discussion

Characteristics of the various ASIC architectures developed for MD are summarized in Table 7. The ASIC designs discussed follow a similar trajectory to FPGA-based development. In the earlier period of the work, the MDGRAPE^118,128,130^ and MD-Engine^120^ designs implemented brute-force nonbonded calculations, even if they were part of a larger more complex simulation workflow as is the case in ref (120). The Anton design implemented the full-md simulation on chip coupled with a more sophisticated PME nonbonded force calculation. Subsequent ASICs followed this design closely, moving to SoC-based designs with PME based nonbonded force calculations.

**Table 7:** Characteristics of ASIC Designs

year	name	alg.	arch.	force calc.	force accum.
1996	MD-GRAPE^128^	direct	nonbond only	32-bit float	80-bit fixed
1999	MD-Engine^120^	direct	nonbond only	40-bit float	64-bit float
2003	MDGRAPE-2^130^	direct	nonbond only	32-bit float	64-bit float
2003	MDGRAPE-3^118^	direct	nonbond only	32-bit float	80-bit fixed
2009	Anton^141^	GSE^34^	full MD	32–36 bit fixed	86-bit fixed
2014	MDGRAPE-4^134^	GSE^34^	full MD	32-bit float	32-bit fixed
2014	Anton 2^140^	*u*-series^142^	full MD	32–36 bit fixed	86-bit fixed
2021	Anton 3^139^	*u*-series^142^	full MD	14–23 bit fixed	14–23 bit fixed

ASIC implementations of MD remain orders of magnitude “faster” than alternative architectures (for example, Tables 8 and 9). ASICs are also orders of magnitude more expensive to build, with the costs for full clusters given in the millions of USD.^118,134^

**Table 8:** Performance of Various Accelerator Configurations to Run a Single Simulation of Dihydrofolate Reductase (DHFR)^a^

accelerator	engine	time scale (ns/day)
Anton 3 (64-node) (ASIC)	Custom^139^	212 200
Anton 2 (512-node) (ASIC)	Custom^140^	85 800
Intel Stratix 10 (FPGA)	Custom^98^	630
2x Nvidia Titan-RTX (GPU)	Amber^154^	629.03
NVIDIA V100 SXM (GPU)	Amber^154^	522.20
NVIDIA V100 PCIE (GPU)	Amber^154^	277.14
NVIDIA TITAN X (GPU)	OpenMM^155^	393
NVIDIA TITAN V (GPU)	OpenMM^155^	419
NVIDIA RTX 3090 (GPU)	ACEMD^156^	1308

a DHFR is a 159-residue protein (suspended in water) target for cancer therapeutics that has been used as a standard benchmark for MD simulation throughput. All simulations reported here employ NVE microcanonical constraints. NVE refers to the set of constraints on MD simulations in which moles (N), volume (V), and energy (E) are conserved in the simulation. All simulations reported here with the exception of Anton 2^140^ use the PME^32^ algorithm for non-bonded interactions. Anton 2 uses the μ-series^142^ algorithm for non-bonded interactions.

**Table 9:** Selected MD Simulations among the Largest of Those Reported in the Literature

name	# atoms	time scale (ns)	resource	engine	year
SARS-CoV-2 viral envelope^78^	304 780 149	84	Summit	NAMD 2.14	2021
H1N1 2009 viral envelope^157^	160 653 271	121.04	Blue Waters	NAMD 2.10	2020
GATA4 gene locus^158^	1 000 000 000	1	Trinity	GENESIS	2019
STMV^159^	1 066 628	13	NCSA Altix	NAMD 2.5	2006

The justifications for development of ASICs have been cited as being a choice between developing better algorithms versus developing specialized hardware.^117,118,120,130^ The large upfront costs were amortized by the expected improvement in potential simulation throughput versus general processors which was demonstrated for refs (40 and 141).

As a consequence of the high cost and technical expertise required, development of MD specific ASICs has also been mostly relegated to two institutions, the Riken Institute funded by the Japanese government and the privately funded D.E. Shaw Research group based in the United States. Thus, access to ASIC-based MD engines remains an issue. In response, the Anton machines^117,140^ have been provided to researchers as part of an NIH grant at the Pittsburgh Supercomputing Center. Recently, MD simulation trajectory data of the SARS-CoV-2 main protease generated by the MDGRAPE-4 series of ASICs has been made publicly available.^135,143^ Additionally, the MDGRAPE-4 series and Anton 2 ASICs have improved their programmability by implementing a C/C++ interface.

The Anton and MDGRAPE series of ASICs have helped make significant strides in the simulations of fundamental biological processes including protein-folding and protein–ligand binding.^135,141,144^ In the face of this, much attention is being shifted toward the integration of machine learning into molecular simulation workflows,^78^ and fundamental research is ongoing in the development of more accurate force fields using deep learning and quantum mechanics.^19,85^ Future development of ASICs will undoubtedly depend on the outcomes of these efforts.

## 7 Future Directions

### 7.1 Heterogeneous Architectures

With increasing complexity of workflows, it is becoming difficult to design a processor which can optimally handle a diverse range of tasks most effectively.^78,90,93,145−150^ Instead, there is acceptance in the industry that competing architectures can be viewed as complementary devices and potentially can be coupled together in a *heterogeneous* architecture. The OneAPI project^151^ is a primary example of the direction the hardware industry will take in the future for design of servers that will come to be used across industry, government, and academia. CPUs excel at instruction level parallelism and are able to handle diverse sets of tasks, GPUs are able to exploit data parallelism with orders of magnitude more cores than a traditional CPU, and FPGAs can approach ASIC performance as they exploit instruction level and data parallelism while being able to be reconfigured thus coming at a lower cost in terms of investment in technical development or cost to acquire. A heterogeneous workflow can ideally map various simulation steps to a specific architecture that are best suited to exploit the optimal performance. Challenges remain in designing tools that can allow developers to design their codes to leverage multiple hardware platforms, which do not all support the same instruction sets.^152^ The AMD Heterogeneous-Computing Interface for Portability (HIP)^153^ is a solution for enabling heterogeneous device development, allowing CUDA-based code to run on NVIDIA and AMD devices. In the meantime, it is of great utility to better develop quantitative studies of how the various architectures covered in this work compare overall as well as specific investigation of the various components of the MD workflow can be mapped optimally among the classes of accelerators (Table 10).

**Table 10:**
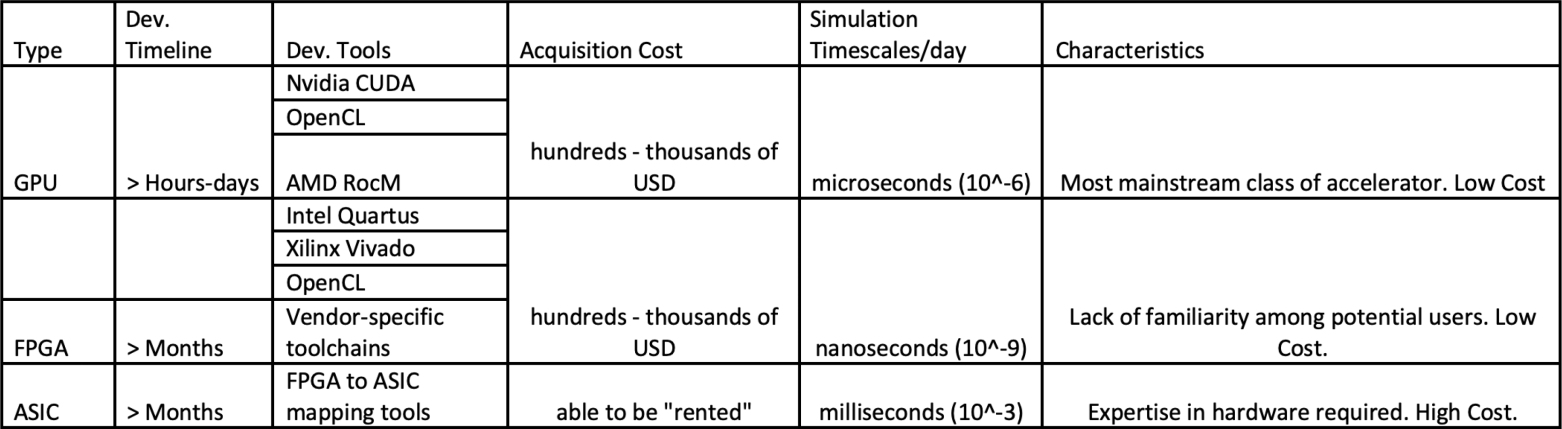
Qualitative Comparison of Accelerator Classes Covered in the Discussion^a^

a The time scales quoted here correspond to results collected for a common benchmark study that has been considered among the various architectures discussed in this work, Dihydrofolate Reductase (DHFR), and are not meant to be presented as definitive assessments.

### 7.2 Synthesis of Machine Learning and Hardware Acceleration for MD

The integration of machine learning and molecular dynamics has picked up much enthusiasm in recent years.^160^ With the rise of machine learning integration into molecular simulation workflows, especially those of deep-learning, present areas of interest for work include demonstrating how these technologies can be used in tandem with hardware accelerators to better exploit molecular dynamics simulations.^161−163^ The JAX, M.D. project^164^ aims to develop a differentiable molecular dynamics workflow by providing a framework that allows for seamless integration of machine learning models with physical simulation code. JAX, MD itself is built upon the JAX python library which integrates Autograd and the Tensorflow accelerated linear algebra library, XLA,^165^ to allow for high performance codes to be deployed on GPUs and Tensor Processing Units (that is, TPUs) which are AI ASICs designed by Google.^166^ The TorchMD python library^85^ was recently introduced and presents another attempt to bridge machine learning and molecular dynamics simulations, providing an interface built upon the PyTorch^167^ deep learning library. By adhering to a PyTorch backend, the TorchMD tools can also leverage hardware accelerators such as GPUs and TPUs, as well as others that may come to be supported by PyTorch in the future. The initial debut of the TorchMD library compared to a production MD simulation code highlights the need for further optimization of the code itself, but also where the utility of more capable acceleration in terms of software design as well as the target hardware architectures themselves are likely to play a crucial role in enabling the practical use of differentiable MD simulations in the future.

Numerous hardware accelerators for AI are coming to market to meet the insatiable compute demand (measured in floating point operations per second or FLOPs) for machine learning workflows, estimated as doubling every 3.5 months.^168^ The Cerebras Systems Inc. CS-1/CS-2 wafer scale processor^169^ and SambaNova Reconfigurable Dataflow Architecture^170^ are recently proposed alternatives to traditional multicore architectures. Cerebras CS-2 is the largest chip ever built, fabricated from the largest square from a single silicon wafer. The CS-2 is over 56× larger than the largest GPU, features 850 000 programmable compute cores, 40 gigabytes of on-chip SRAM, 20 petabytes/sec of memory bandwidth, and 220 petabits/sec of interconnect bandwidth. The system is additionally supported by custom compilation tools that optimize the reconfigurable dataflow processing units depending on the users application. Cerebras also provides support for Tensorflow and PyTorch APIs, drastically lowering the barrier to entry. The SambaNova DataFlow Accelerator is motivated by a similar use case in machine learning acceleration with its own reconfigurable dataflow architecture, custom compilation tools, and custom “SambaFlow” software stack supporting Tensorflow and PyTorch APIs. The Graphcore Intelligence Processing Unit (IPU) is yet another AI accelerator poised to challenge the status quo of the GPU-centric development environments used in AI and MD simulations today.^171^ As these and likely additional accelerators come to market, offering advantageous levels of computation relative to energy utilization, the current status quo of a GPU-centric development environment is likely to pivot to utilize these architectures with tight integration with differentiable machine learning libraries.

### 7.3 Further Improvement of Accuracy and Algorithms

The discussion covered in this work considers the context of NVE-ensemble simulations which have been implemented across all of the various architectures employed. It is also the case that simple integration algorithms such as velocity Verlet are mostly common among the works discussed. With that said, research into improving the accuracy as well as efficiency of MD simulations remains an active area so it is important to highlight some of the work as future systems in heterogeneous environments will need to be designed with such features in mind.

An obvious limitation of the classical MD algorithm as described in this review is the order of the integration time step, which is often chosen as 2 fs in order to capture the most granular behavior, bond vibrations as shown in Figure 1. One technique featured in modern MD engines is Hydrogen Mass Repartitioning (HMR) which allows for the integration time step to be increased by a factor of 2 with minimal loss in accuracy or stability of the simulation trajectory by repartitioning the mass of heavy atoms into the bonded hydrogen atoms.^172^ Other techniques for improving the efficiency of MD simulations include Multiple time scale MD,^45^ which partitions the interacting sets of atom neighbors into *primary* and *secondary* interactions, and then update force calculations at staggered time intervals. Additionally, Reaction-Field Electrostatics^173^ is another method to improve the efficiency of long-range electrostatics and is implemented in popular MD packages such as OpenMM.^174^

Other limitations of the MD algorithms covered in this work are the empirical force fields that are employed in the simulations. Ab initio molecular dynamics (AIMD) treats the electronic structure of the atoms in the simulation explicitly and can provide more accurate description of the dynamics of the system (for example, bond formation and breaking).^175^ While AIMD has historically been prohibitively expensive to consider for the systems covered in this work, progress has been made in the acceleration of these calculations using deep learning to approximate the interatomic potential energy surface from AIMD simulations.^176−178^ A recent work demonstrates the application of a heterogeneous computation system (Summit Supercomputer at Oak Ridge National Laboratory) to scale deep-learning based Potential Energy Surface (PES) models in tandem with the LAMMPS MD engine^179^ to perform studies of very large systems, on the order of 100 million atoms, at the nanosecond time scale with ab initio accuracy.^180^

Much of the MD acceleration covered in this work has been concerned with accelerating the time scales of the microscopic kinetics of the biomolecules ranging from very short time scales to what are hopefully biologically meaningful time scales. However, even a trajectory extracted from a relatively long-time scale MD simulation is susceptible to becoming “stuck” in one of the huge numbers of energy minima in the high-dimensional potential energy surface due to the presence of high potential energy barriers between these minima.^181,182^ Lacking an ability to effective sample low-energy states of a biomolecule negatively affects the accuracy of the macroscopic thermodynamic properties such as free energy, a critical measurement especially for applications in drug discovery. Modern MD packages include support for algorithms that allow for improved sampling such as Replica-Exchange Molecular Dynamics (REMD),^181^ Metadynamics (MTD),^182^ and Adaptively Biased MD (ABMD).^183^

Enhanced sampling methods are provided in packages such as PLUMED,^184,185^ which wraps existing MD simulation engines such as those discussed in Section 4.2. GPU packages such as Amber also provide support for enhanced sampling algorithms.^75,81^ Anton also has been shown to implement enhanced sampling algorithms,^186−188^ and the latest iteration of Anton suggested continued development for enhanced sampling methods.^139^ Some of the challenges in implementing these types of algorithms in specialized hardware have been identified previously.^186^ Specifically, the memory resource requirements for replica exchange (RE)^181^ forced the Anton 2 machine to use multiple Anton nodes to run a single replica of a typical solvated protein.^186^ Considering that REMD may require hundreds of replicas,^80^ the Anton architecture was seen as being better suited for the simpler Simulated Tempering (ST) algorithm,^189^ where the entire machine could better accelerate a single simulation.^186^ Additionally, REMD imposes communication requirements when exchanging systems between nodes. The frequency of exchanges is a parameter that may be tuned, but as the exchanges are attempted more frequently, the communication burden grows. The current generation of Anton (3) is presumed to have greater amounts of SRAM available, given the current description of the machine features an increase of nearly 2 orders of magnitude capacity (atoms) per node.^139^ Enhanced sampling may also require other functions in addition to the force field to be evaluated. ST requires evaluation of the potential energy function,^186^ which at the hardware level required updating force calculation parameters to instead compute energies. Lastly, while an FPGA-focused implementation of an enhanced sampling algorithm has not been presented in the literature, ongoing work that studies networks of FPGAs for MD acceleration could potentially be extended to enhanced sampling algorithms.^190^ Challenges faced by the Anton machine are likely applicable to the case of FPGAs, such as dynamically swapping between evaluation of forces and potential energy. Additional work in applying machine learning to accelerate sampling of atomic systems is ongoing with promising results.^191,192^

## 8 Conclusions

We have reviewed three broad categories of accelerators for molecular dynamics simulations that include GPUs, FPGAs, and ASICs. Our discussion details the benefits of rich development environments that have enabled the popularity of GPUs for MD acceleration while also identifying outstanding issues of the hardware itself to scale popular long-range electrostatics algorithms effectively. We then investigated the history of MD acceleration in the context of FPGAs, which exhibit favorable properties such as flexibility in data types in contrast to GPUs, elimination of control and synchronization overheads, while exhibiting instruction-level and data-parallelism. Despite these properties, however, a major hurdle in the successful mainstream adoption of FPGAs as accelerators for MD has been a lack of accessible development tools for researchers. That stated, FPGA hardware as well as development tools have progressed significantly since the first MD applications were developed in the previous decade, and industry trends suggest a future in which servers will become more heterogeneous. Today there are already examples of servers featuring FPGAs and microprocessors on the same chip as well as the top supercomputers in the world regularly featuring GPU coprocessors to accelerate a diverse array of workloads that often combine MD with machine learning and deep learning. While ASIC architectures have historically been cost-effective to reduce the “time to solution” for achieving biologically relevant MD time scales, they are also expensive to develop and lack the flexibility to be reprogrammed to a significant degree. The GPU has been enormously beneficial to the scientific community for its ability to democratize longer-time scale MD, as well as fuel research into more effective MD sampling methods and thermodynamic property calculations. With more specialized architectures for AI appearing, such as the Cerebras Wafer-scale accelerators, SambaNova Reconfigurable DataFlow architecture, and Graphcore Intelligence Processing Unit (IPU), it is not difficult to imagine a future where MD applications which are already beginning to feature tight integration with machine learning and deep learning will be designed to leverage a diverse array of processing capabilities rather than being implemented entirely on one paradigm.
